# Understanding decision-making around human and livestock health in sub-Saharan Africa: A systematic literature review

**DOI:** 10.1016/j.dialog.2025.100259

**Published:** 2025-12

**Authors:** Mary Nthambi, Tiziana Lembo, Alicia Davis, Blandina T. Mmbaga, Nicholas Hanley

**Affiliations:** a The Boyd Orr Centre for Population and Ecosystem Health, School of Biodiversity, One Health & Veterinary Medicine, College of Medical, Veterinary & Life Sciences, University of Glasgow, Glasgow, UK; b School of Social & Political Sciences/School of Health & Wellbeing, University of Glasgow, Glasgow, UK; c Kilimanjaro Clinical Research Institute, Moshi, Tanzania; d Kilimanjaro Christian Medical University College, Moshi, Tanzania

**Keywords:** Decision-making, Human health, Livestock health, Inequalities, Gender, Household production of health, One health, Trade-offs

## Abstract

Household decisions shape health outcomes in subsistence farming communities in sub-Saharan Africa (SSA) where human wellbeing is closely linked to livestock productivity. We conducted a systematic review, guided by Preferred Reporting Items for Systematic Reviews and Meta-Analyses (PRISMA), of 135 primary studies from Embase, Scopus, PubMed, Web of Science and Google Scholar. Using the Household Production of Health (HPH) framework, we structured research questions, extracted and synthesised evidence and identified health interventions in human and livestock health in SSA. We coded decision loci (sole vs joint decision-making) and characterised their prevalence, context and determinants of household health outcomes. Sole decisions dominated (40 % of human studies, 42 % of livestock studies) with men making the majority of the decisions, especially in livestock health, while joint decisions were less common (27 %, 32 % respectively) and focused on maternal, neonatal and child health. Women's decision-making power tended to increase with education, income and urban residence, while male authority was greater in rural areas where fewer income-earning opportunities for women prevail. The two HPH frameworks are tightly linked as behaviours that improve livestock health and productivity benefit nutrition, income and access to care for household members. We recommend gender-responsive, One Health policies that support women's control in areas of existing responsibility and engage men as active partners in shared decision-making within households to reduce inequalities. These efforts should be supported by progress towards universal health coverage, functioning health insurance schemes and accessible veterinary services to reduce inequalities and within-household trade-offs, and to improve health and livelihood resilience in SSA.

## 1 Introduction

Subsistence farming communities in sub-Saharan Africa (SSA) face high levels of morbidity and mortality in humans and livestock due to the burden of infectious and non-infectious diseases, and malnutrition. Deaths resulting from human diseases such as malaria, tuberculosis, human immunodeficiency virus infection/acquired immune deficiency syndrome (HIV-AIDS), diabetes, cancer and cardiovascular diseases are common. For example, SSA accounts for about 29 % of tuberculosis [1], 69 % of HIV-AIDS [2] and 93 % of malaria [3] cases in the world. Non-communicable diseases are also rising and, according to recent projections, are estimated to cause 27 % of deaths [4]. Livestock production losses resulting from high disease burdens are common, harming regional and national economies, reducing household income and wealth, especially in impoverished communities. In the region, livestock contributions to the economy amount to about 25 % of gross domestic product (GDP) [5]. At the household level, livestock provide food and income and are held as a store of wealth [6]. Here, livestock are not only important agricultural assets but also a symbol of prestige, culture and religion [7]. Livestock diseases threaten this socioeconomic value. For instance, contagious bovine pleuropneumonia (CBPP) causes an average annual income loss of around $3933 per household - roughly 232 % of the net income from cattle ($1697) in Narok District, Kenya [8]. Of the losses associated with CBPP, disease reporting and treatment account for about 34.7 % and 48.4 % of the annual losses [8]. Foot-and-mouth disease (FMD) causes direct production losses of $2.3 billion/year in Africa [9] - over 0.001 % of SSA's entire GDP [10] - and it restricts national and household economies [11]. Further losses associated with livestock keeping may be connected to human disease arising from zoonoses (i.e. diseases transmitted between animals and humans) [12]. As such, livestock disease causes human suffering, loss of identity and cultural autonomy, and affects personal relationships between people and their animals [13].

Understanding household decision-making in human and livestock health helps identify interventions that reduce infections and associated costs (e.g. morbidity and mortality) and improve human and livestock health outcomes. There are several existing studies (e.g. [[14], [15], [16], [17]]) on decisions around access to health facilities, reproductive health, nutrition, health investment and expenditure, as well as treatment and prevention of diseases in humans and livestock in SSA region. However, few studies show how health is “produced”, and who “consumes” or benefits from it within the household, considering both economic and broader (e.g. socio-cultural) perspectives. Most studies show that decision-making related to human health across SSA is characterised by gender biases arising from household authority structures (e.g. [[18], [19], [20]]). Although joint decision-making is often cited as an important aspect of equitable health outcomes, there remains limited understanding of how joint decisions are made in practice across different household health domains. Existing research tends to focus on single health concerns, e.g. family planning, without adequately capturing factors influencing overall household decision-making (e.g. [[21], [22], [23]]). Few existing studies integrate livestock health into the household health narrative, despite its role in shaping nutrition, income and within-household resource trade-offs that directly affect human well-being. This underscores the need for a more integrated, gender-sensitive and comprehensive “One Health” analysis and understanding of household decision-making in the SSA region.

To understand the situation further, we start by defining a *household* as the most basic economic unit around which decision-making and well-being can be understood [24]. While Becker's [25] household production model provides a foundational economic framework for analysing household decisions, its assumption of a single decision-maker limits its applicability in many real-world contexts. We, therefore, adopt a broader, collective perspective [24,26] that recognises intra-household dynamics, negotiation and joint decision-making. This aligns with the Household Production of Health framework [27], which emphasises how households combine internal resources and external inputs to maintain and improve health outcomes. We apply this framework to explore decision-making in livestock and human health in the SSA region, informed by both economic theory and socio-cultural context.

The “Household Production of Health” (HPH) framework is defined as a behavioural process in which households combine social, economic and health inputs to “produce” and maintain desired health among household members [27].

HPH is a single comprehensive framework that brings together several approaches to describe health problems in the sociocultural and economic environments [27]. Placing the household at the centre of health outcomes, the HPH framework explains the behaviour of individuals resulting from their interaction with the social, economic and health system settings within which the household operates. However, we recognise that health spans beyond economic factors and encompasses biophysical, spiritual, emotional and cultural aspects of life, as a household is made up of people with different behaviours and personalities. We extend the HPH framework to include livestock as a production asset and identify the shared determinants of health outcomes at the household level. Furthermore, gender dynamics are cross-sectoral as men's control over livestock assets affect women's ability to act on maternal and child health decisions and effectively meet household nutrition needs. As a result, integrated evidence is important to support One Health and universal health care policy designs.

We assume households comprise individuals whose behaviours and choices produce health outcomes, and who also demand and value those outcomes. Health production within the household occurs through decision-making related to disease treatment and prevention. Health decision-making expands beyond issues centred around disease, as health is more than just the absence of disease [13,28]. However, a focus on disease prevention and treatment is valuable in this setting where structural issues around hygiene, inequalities and infrastructure are common [29]. The framework recognises that members can produce health together or individually using household resources. Produced health is then available to, and valued by, any household member.

The HPH framework has proven valuable in a range of contexts in both developing and developed countries. For example, it has been used [30] to evaluate the impact of Buruli ulcer in Benin, West Africa and to explain the link between marriage and good health status [31]. Moreover, the HPH framework enabled the identification of determinants of neonatal mortality in Iran [32] and the functionality of elderly people in the USA [33]. However, our use of the HPH framework is novel as we systematically synthesise evidence on decisions around the sole and *joint* production of human and livestock health across SSA. Our approach identifies the inputs into separate human and livestock health production frameworks and then establishes the relationship between them. We show how health producers, and those that demand health, interact with the environment (the social, economic and health systems). This helps in identifying gender and intra-household inequalities in resource allocation. It also allows us to show that some of these disparities may be mitigated through shared decision-making, enabling men to act as allies to women within the household. Overall, we compare sole and joint decision-making across human and livestock health, mapping their prevalence, context and links to resource allocation and health outcomes in SSA. This is critical to demonstrating the value of linking resource allocation to health in areas where demand and need are high. To the best of our knowledge, no previous study analyses household decision-making using the HPH framework in the same depth for this region.

### 1.1 Household, family and gender roles in sub-Saharan Africa

In Africa, most economic activities still take place within traditional settings (i.e. peasant and less stratified societies with highly socialised households) characterised by a sociocultural dimension, an understanding of which is important in the analysis of data and the interpretation of research [34]. We use a standard definition when referring to the term “household”, as who makes production and consumption decisions depends mainly on culture and composition of the household. In this paper, a traditional household is characterised by a group of people living within the same compound, who are likely related to each other through blood or marriage, who share meals and pool resources or make income-related decisions jointly [35]. Furthermore, in a household in rural areas of the SSA region, it is common to find a household head living and working away from his wife and children but still participating in major decisions in the household.

According to the United Nations [36], the definition of a household should be flexible and be based on the household size, composition and socioeconomic processes such as childbirth (population growth), demand for health care, housing and schooling and setting of priority on expenditure needs. Household structure is influenced by marriage type, the number of children, intergenerational norms, employment type and costs of housing among other factors [36]. Anthropologists have long explored marriage in relation to lineage, with a long history of exploration of marriage and household systems across Africa. Across many African contexts, marriages are organised patrilineally (a woman moves to her husband's home) or matrilineally (a man moves to his wives' home) [37]. In matrilineal marriage, mothers own children and, traditionally, women have high decision-making power, while in patrilineal marriage fathers own children and men have the final say on decisions [38].

It is worth noting that the terms “household” and “family” are not synonymous. A household is a geographic/economic unit made up of individuals who share a common residence (e.g. a house, *boma* and production base), a shared resource pool and who participate in joint production and consumption decisions [31,36]. In contrast, a family is a kinship unit extending across multiple households, consisting of individuals who are biologically (e.g. brothers, sisters, parents, children) and non-biologically (e.g. in-laws, age-mates) related, and who maintain social, economic and emotional ties that strengthen their collective identity. A household as an institution goes beyond the production and consumption processes to include the social and political order which, to a larger extent, manifests in the context of intra and inter-household interactions [27,39].

Members of a household are governed by a set of rules and informal agreements that define their behaviour and gender roles. For instance, most societal norms across SSA place men in the highest position of society such as a community leader, the primary decision-maker, the breadwinner or the head of the household and income provider [40]. Women are mainly caregivers or home-keepers [40], responsible for domestic activities [41] such as feeding children, matters involving sanitation and health-seeking activities [42] and fetching water and firewood [43]. However, this characterisation is restrictive and does not acknowledge the other critical but often undervalued roles women play in sub-Saharan African economies. For instance, in many parts of West Africa, women engage in trade and agri-business opportunities and, therefore, contribute to the generation of income [44]. Similarly, in East Africa, women play an important role in the agricultural workforce as livestock keepers, especially of small stock and farmers, which has empowered them to engage in decision-making in the household, although men still control most high-income generating activities [45]. These regional variations highlight the importance of not generalising gender roles across diverse African contexts. Additionally, colonial histories influenced gender roles, often reinforcing patriarchal structures [46]. Furthermore, feminist movements across the SSA region have consistently sought to modernise and diversify gender roles, addressing economic and political dimensions that shape these roles [47] for equitable access to income-generating activities [41].

## 2 Critiques of the household production of health (HPH) framework

In the mid-1990s, Berman et al. [27] argued that most public health programmes in developing countries focused on solutions to specific diseases while overlooking the broader household environment in which health decisions and choices are made. The authors proposed the HPH framework as an integrative model drawing on economic models and grounded approaches in anthropology [27]. At the time, their discussion was limited to the production of health which the authors acknowledged fell short of fully addressing all the expectations of social scientists (e.g. economists, anthropologists, psychologists etc.) and epidemiologists. Given this limited scope, they identified four areas requiring further research to broaden the framework's applicability. The first area includes household dynamics, its structure and functions, and how these influence treatment-seeking behaviours and, ultimately, health outcomes. A second aspect is how individuals perceive and define ill health, including how intra-household processes interact with the health-care systems. Thirdly, health-producing and health-sustaining behaviours within households and their effects on individual health need to be considered. Finally, the factors that influence health-producing behaviours and their relationship to the household's general welfare are important.

Berman et al. [27] also recognised that the HPH framework might offer limited practical guidance for health interventions at the community level. Aware of this, Pattanayak and Wendland [48] cautioned that aggregating health determinants at community level risks information loss through an overly narrow focus. They proposed aggregating determinants at the individual and household levels, and grouping them into three categories: direct factors (e.g. nutrition and health care); indirect and mediating factors (e.g. socioeconomic and cultural factors); and linking factors that connect the direct and indirect factors (e.g. maternal health).

Perhaps reflecting these limitations, Berman et al.'s [27] framework saw limited uptake in public health matters until 2001, when Simon et al. [49] reiterated health production as a function of the family system. A family is a group of people, not necessarily biologically related, who assume the responsibility for an individual's health in ways consistent with recognised kinship and household matters [49]. Similarly, Crandall et al. [50] highlighted the central role of families and their settings in public health, emphasising that families help produce health, encourage healthy choices and support behaviour change in policy and interventions [50]. The underlying assumption is that interactions within family relationships shape decisions [51]. Some family members combine knowledge, attitudes and behavioural norms with social factors, such as age, income and education among others, to make health decisions [52]. Others, particularly children and older adults - including those with chronic conditions or mental health issues - primarily require care and are likely to benefit from, rather than contribute to, health-related decision-making at the family level [50].

### 2.1 Application of the HPH framework in our study context

We use the HPH framework to structure our review and define health outcomes from two perspectives, depending on whether the focus is on the internal household processes (production and consumption decisions) or external processes (institutional approach described by the sociocultural, economic and health system context). For internal processes, we examine the locus of decision-making and identify who and how many people participate in decision-making (sole or joint), following approaches used in Acosta et al. [53] and Bernard et al. [54]. These authors classify households by the number of decision-makers (sole or joint) involved in production, consumption and decisions about financial resources in agricultural settings [53,54]. For the external processes, we treat the household itself as an institution comprising decision rules, norms and bargaining processes that affect the way it operates [27]. In this regard, Berman et al. [27] identify three components: 1) the social; 2) the economic; and 3) the health-system environments. In this study, we add a fourth component that encompasses individual- and household-level characteristics largely associated with demographic factors.

First, we distinguish between sole and joint decision-making. In the sole decision-making processes, a single individual makes decisions with minimal or no input from other household members, whereas in joint decision-making, spouses or multiple members decide together [54]. Under sole decision-making process, the dominant individual makes choices subject to budget and time constraints [see 27,31]. The model assumes pooled income regardless of who earns it and decides for all household members [54,55]. Such models do not account for unequal decision-making power or disparities in ownership of resources and income [55] arising from gender inequalities and patriarchal or familial hierarchies, including norms linked to ancestry and religion.

By contrast, in joint decision-making, household members bargain, allocate resources and contribute to choices to achieve collectively desired outcomes [54,56] (for bargaining models, see [57,58]). Joint resource allocation can enhance efficiency by incorporating diverse preferences and information [31], though in some instances, sole decision-making may be more effective, depending on the choice at hand. Nonetheless, joint decision-making often increases the aggregate satisfaction of household members compared to sole decision-making.

The HPH framework is well-suited for providing conceptual perspectives as in Agbo et al. [30] and Tipper [31] (see its mathematical formulation in Supplementary material 1). We use it to describe health decision-making within households under the following assumptions: (1) households engage in behaviours to produce health, through decisions about disease treatment and prevention (2) major decision makers in the households are health producers, while those for whom decisions are made are receivers of health; (3) health producers operate within social, economic and health system contexts that are context-specific to each household, community or broader environment; and (4) health producers make decisions based on how they value those who demand health, the resources at their disposal, their gender, bargaining power, skills or education, and the time available to them, among other factors. We acknowledge that these assumptions may not fully account for the wide range of complexities associated with these household processes. However, we find the framework useful to extract evidence, map findings and suggest interventions on livestock and human health in SSA.

## 3 Literature search

### 3.1 Sources of data and search terms

We searched and reviewed the literature following PRISMA (Preferred Reporting Items for Systematic reviews and Meta-Analyses) guidelines for systematic reviews [59]. We focused on primary studies from five databases, namely Embase, Scopus, PubMed, Web of Science and Google Scholar. We restricted ourselves to articles with the following search terms and Boolean combinations: ((Household) AND ((decision-making) OR (choice experiment) OR (choices) OR (financial resource allocation) OR (resource allocation) OR (health) OR (gender roles) OR (decision-making power) OR (livestock) OR (livestock decisions) OR (health care expenditure decisions) or (out-of-pocket expenditure)) AND health) in sub-Saharan Africa OR Africa. These search terms were agreed upon and discussed among all authors at the start of the review process and were uniformly applied in all selected databases. Snowballing was also applied to obtain any additional relevant references within the bibliography of selected studies.

### 3.2 Study inclusion and exclusion criteria

The studies we selected met the inclusion and exclusion criteria agreed by all authors. Any disagreements were resolved through discussion. Firstly, household decision-making, choices, gender, resource allocation and out-of-pocket expenditure had to be explicitly addressed in the primary studies. Secondly, studies had to be based on primary data collected through methods such as surveys, in-depth interviews, visualisations or focus group discussions involving households, community members, or health providers in rural or urban areas of any country within SSA. Thirdly, studies had to have been published in the English language after January 2000 and by May 2020. We set this search window to maximise comparability and review quality as, from 2000 onwards, household decision frameworks, One Health policy design and implementation, and gender-sensitive measures became more standardised in the SSA region, providing a larger literature for extraction and appraisal (e.g. using the Newcastle-Ottawa scale). Furthermore, because the review process took place during the COVID-19 pandemic, we retained the May 2020 cut-off to ensure a consistent pre-pandemic household decision environment. Fourthly, eligible decisions had to be in the areas of human and/or livestock health, e.g. nutrition, feeding, maternal health care and child delivery, health expenditure etc. We excluded systematic reviews, studies not focusing on decision-making at household level (e.g. teenage pregnancies in schools), studies not from a country in the SSA region or those unrelated to health in human and livestock. We also excluded studies that were conducted outside the range of our time frame, i.e. January 2000-May 2020. All included studies used original empirical data and reported identifiable approaches for assessing household decision-making dynamics.

### 3.3 Data extraction

**Fig. 1:**
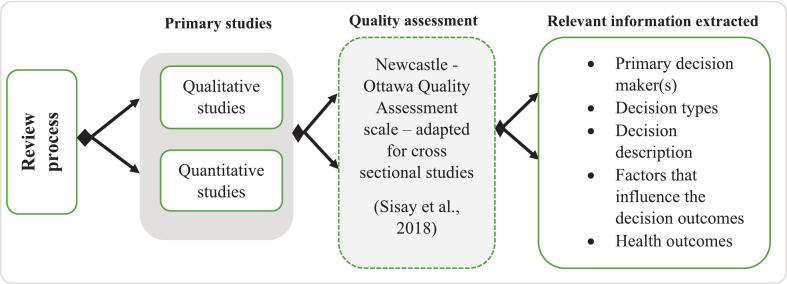
A diagram showing the three main steps of the review process in this study and information extracted.

Studies selected were first screened by title in all databases and the relevant ones were exported to the Endnote online software (Clarivate in Web of Science). Duplicates were removed from the Endnote online software and abstracts were screened for relevance to decision-making and resource allocation in health. Studies that met the predefined eligibility criteria agreed upon by four authors were moved to a separate folder in the Endnote software for in-depth review of the full text and information extraction. The review comprised two steps: (i) the first author conducted a comprehensive full-text review using the production of health approach and recorded the study details in an Excel sheet (Supplementary material 2), resolving any disagreements or ambiguities through discussion with the other authors, and (ii) we then assessed the methodological quality of the eligible qualitative or quantitative studies selected and extracted information (see Fig. 1). Studies that met the quality criteria were retained (Supplementary material 2). Included studies were grouped into themes and recorded in the spreadsheet for further evaluation and interpretation by all authors (Supplementary material 2).

### 3.4 Quality assessment of selected studies

We used the Newcastle-Ottawa Quality Assessment Scale (NOS), adapted for cross-sectional studies, to rate each included study on a 10-star scale. Following Sisay et al. [60], we applied three quality criteria for cross-sectional studies. The first indicator assesses methodological quality up to a maximum of five stars. The second indicator assesses the degree of comparability up to a maximum of two stars. The third indicator assesses outcome evaluation including independent blind review, and the clarity and appropriateness of statistical analyses (for quantitative studies) with a maximum of three stars. Consistent with Desyibelew and Dadi [61], studies with an overall score of ≥6 stars were considered of sufficient quality for inclusion.

### 3.5 Literature synthesis

From the selected studies, we assessed what decisions were made, by whom, how and why they were made, for whom they were made and the factors influencing decision-makers. Themes across studies were manually synthesised to develop interpretations linking household decision-making and resource allocation to health outcomes. We reviewed whether decision-making involved one or multiple family members as described in the original studies based on which we classified decisions as joint, sole or unclear. We looked for terms such as “joint”, “shared”, “couples”, “partner”, “spouse”, “communication”, “dialogue”, “discussion”, “consultation”, “bargaining/bargained” or “negotiating/negotiations”. Additionally, we searched for terms like “sole”, “alone”, “authority”, “control”, “approval”, “final say”, “permission”, “single individual” or “one person” to capture instances of one person's dominance in making household decisions. In studies where the language used was not specific about who was involved, we classified the decision-making process as unclear. To ensure reproducibility and reliability, we implemented a rule-based classifier in R using the phrase list (above) to predict each decision type as sole, joint or unclear. We compared the resulting predictions with our manual classifications (see Supplementary material 2) and quantified levels of agreement as a percentage and the Cohen's k using the inter-rater reliability (irr) package in R [62,63]. We also reported class-wise precision, recall and F1 scores (precision-recall metric) to evaluate predicted classes with our manual labels. To support these classifications, we also considered each study's design and the predefined criteria for each decision type.

## 4 Results

**Fig. 2:**
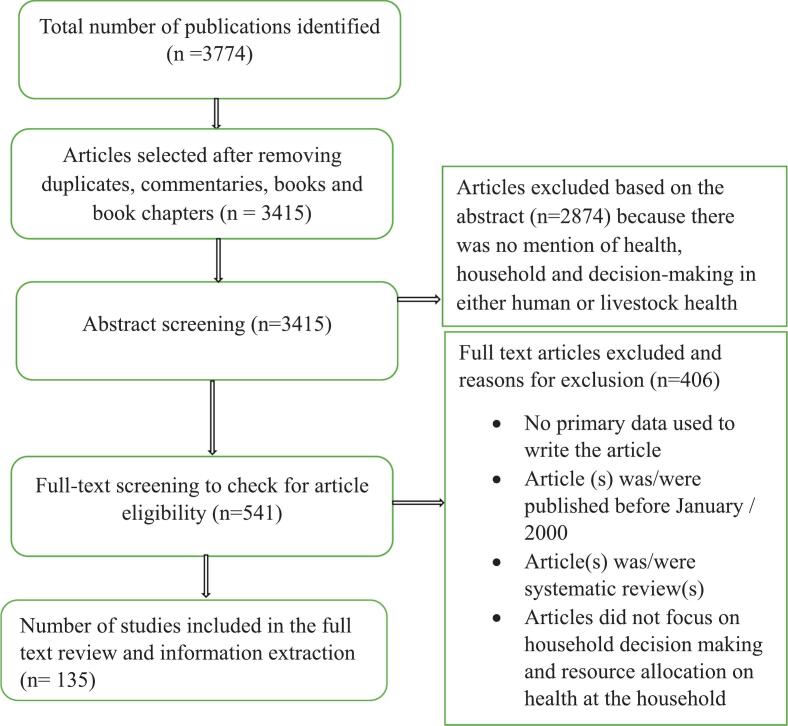
PRISMA flow chart showing the number of studies included and excluded at various stages of the review process.

We identified a total of 3774 articles from the five databases using the search strategy described above (Fig. 2), out of which 135 fulfilled the inclusion criteria.

### 4.1 Characteristics of the selected studies

**Fig. 3:**
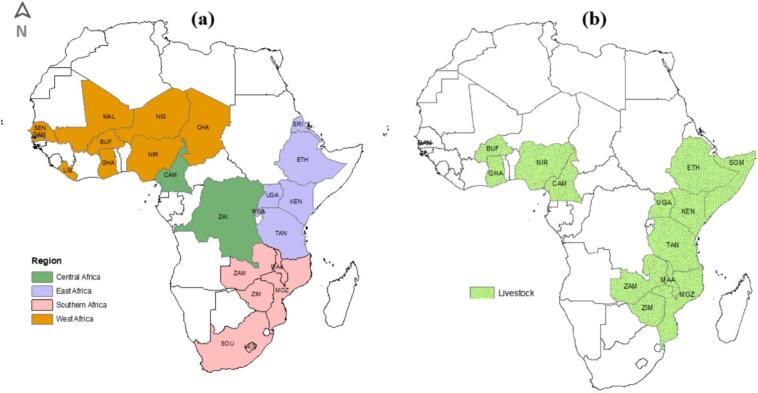
Spatial distribution of countries where the studies relating to health decision-making in (a) humans and (b) livestock included in this review were conducted.

**Fig. 4:**
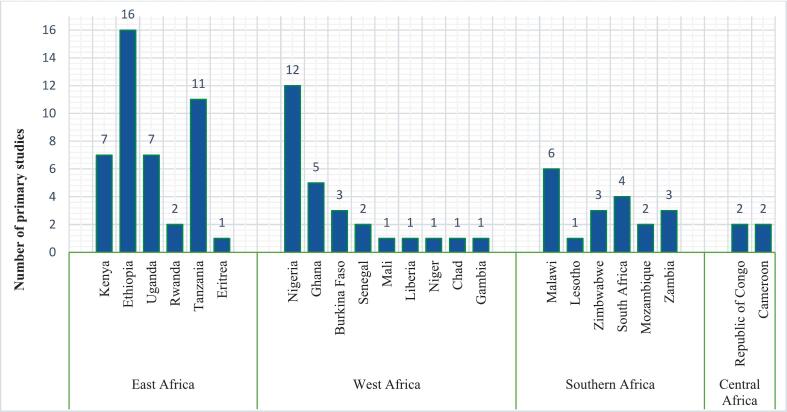
Number of primary studies on decision-making related to human health by country included in the review we describe and discuss in this article.

Here, we highlight some of the key features of the selected studies (for more details see Supplementary material 2). The spatial distribution of countries where the studies were carried out is shown in Fig. 3 (a) and (b). Approximately 70 % (94) of the studies were related to human health and 30 % (41) to livestock health. Human health studies reviewed were from 24 countries in the SSA region. The majority were from East Africa followed by West, southern and central Africa (Fig. 4).

**Fig. 5:**
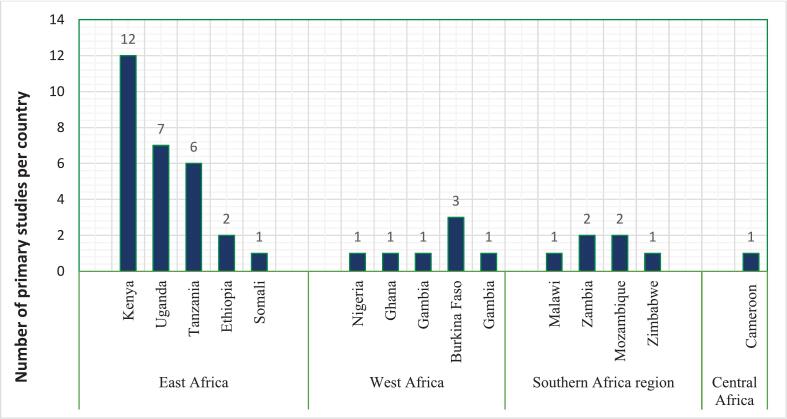
Number of primary studies on decision-making related to livestock health by country we describe and include in this review.

Studies describing livestock health decisions (Fig. 5) were from 15 countries, predominantly within East Africa followed by West Africa and southern Africa. Only one study was from central Africa.

Most studies used data collected in rural and mixed rural-urban settings (Table 1). Urban and peri-urban settings were more common in human health studies than in livestock health studies. We identified nine decision types for human health and ten for livestock health (Table 2). Six types of decisions were common across humans and livestock contexts: disease prevention, treatment, household expenditure (e.g. school fees and food), nutrition, health investment (e.g. health insurance) and health expenditure (i.e. out-of-pocket health care costs). Decision-making regarding maternal health care, childbirth, contraception, fertility and decision-making power was exclusive to human health. Breeding, livestock ownership and decisions around market and resource (income and assets) transfer were exclusive to livestock.

**Table 1:** Location (rural or urban) where the studies reviewed in this paper were conducted.

Place of residence	Human health % studies (*N* = 94)	Livestock health % studies (*N* = 41)
Rural	51	83
Rural and urban	34	10
Urban	4	2
Rural and peri-urban	4	2
Urban and peri-urban	2	–
Urban, rural and peri-urban	2	–
Peri-urban	2	2

**Table 2:** Types of decisions identified in studies on decision-making related to human and livestock health we reviewed for this article.

Human health		Livestock health	
Decision types	% studies (N = 94)	Decision types	% studies (N = 41)
Disease prevention	10	Disease prevention	22
Disease treatment	26	Disease treatment	12
Contraception and fertility	18	Breeding	20
Household expenditure	1	Household expenditure	2
Maternal health care and childbirth	17	Livestock ownership	15
Nutrition	9	Nutrition	2
Health investment	3	Market	12
Decision-making power	4	Health investment	2
–	–	Resource transfer	10
Health expenditure	13	Health expenditure	2

We further classified these decisions as either sole, joint or unclear. We defined sole decisions as those made by a single individual, often a male household head, who exercises dominant authority, either openly or through concealed forms of power

(e.g., requiring women to seek permission or approval) rooted in gendered control over resources (e.g. [[64], [65], [66]]). In contrast, joint decisions were those in which both partners or two or more people actively took part in decision-making through bargaining, negotiation and shared resources, e.g. “shared” spousal decisions on family planning or child health care (e.g. [[67], [68], [69], [70]]). In some studies (e.g. [[71], [72], [73]]), decisions were classified as unclear, due to insufficient details in decision-making. For our automated predictions of classes of decisions, Cohen's kappa (k) indicated almost perfect agreement [74] with our manual classifications. For the human health studies (*n* = 94), overall agreement was 91.5 % (k = 0.869; *p* < 0.001) with class-specific k values of 0.849 (sole), 0.858 (joint) and 0.902 (unclear). For livestock health studies (*n* = 41), overall agreement was 92.7 % (k = 0.887; p < 0.001) and class-specific k values were 0.853 (sole), 0.820 (joint) and 1.000 (unclear). Across all categories, class-wise F1 scores exceeded 0.70, indicating a good predictive performance.

### 4.2 The HPH framework in human health

In 69 % of the 135 studies reviewed, decision-making was classified as either sole or joint. In 31 % of the studies, the decision type was marked as “unclear”, because the main decision-maker(s) could not be determined from the available information. These studies were nonetheless retained for their relevance to the institutional approach to health decisions, whereby choices reflect not only household roles and status but also social, economic and health system contexts.

#### 4.2.1 Sole decision-making

The largest category was sole decision-making (40 % of studies) in human health. Within these, 30 % of sole-decision studies involved male decision-makers and 3 % involved female decision-makers. This suggests that many human health choices are dominated by an individual decision-maker, who has the final say. For example, men/husbands may dictate the contraceptive method their wives should use, which women are expected to accept without question [75]. However, some women still seek contraception without their husbands' knowledge, although we identified differences between rural and urban settings. In rural areas, women who use contraception without spousal approval may be described as acting ‘covertly’ [65], and such behaviour can be framed as an indicator of adultery [75]. In contrast, women in urban areas may express their preferences more freely [75,76].

Men/husbands rarely use contraception to achieve a preferred family size, except when instructed by a health care provider (HCP) to use condoms, for example, to prevent HIV infection or reinfection [77]. An HCP typically refers to a formally trained professional providing care, e.g. treatment, diagnosis, and medicines in hospitals, clinics or community settings [78]. In some studies, HCP also encompassed pharmacy attendants, dispensers and shopkeepers and informal vendors [78]. HCPs make joint decisions with patients considering patients' health problems, choices and expectations. HCPs provide advice and evidence and together with patients, develop an action plan [79].

Another example concerns treatment decisions for newborns and children. Mothers choose their preferred medicine sources to treat febrile illness in newborns. Here, we use ‘febrile illness’ to refer to a new-born syndrome characterised by high fever, catarrh, loss of appetite and coughing [80]. The sources of medicines can either be a shop, hospital or traditional healer [73,81], subject to the household's financial resources [82]. Sometimes a child's grandmother chooses the treatment options which can either be a visit to a hospital, traditional healer or a religious leader [83]. For malaria treatment-seeking, the child's father or the oldest male individual in the household often decides on what care to seek [84].

Maternal health care and childbirth decisions are often made by husbands or grandmothers of sick children. Many pregnant women do not get to choose how and where they access health care. Instead, husbands decide the type of facility, timing of visits and the transport [85]. Husbands also often determine when their pregnant wives seek medical care [85] and sometimes accompany them to antenatal visits for HIV tests [86] and hospitals to provide support, e.g. medical supply purchases [20]. In some cases, paternal grandmothers make choices for first-time mothers during the period of pregnancy, after childbirth and when seeking traditional treatment [87].

Women typically make disease prevention decisions for under-five children [64]. For instance, they determine HIV testing preferences to prevent mother-child transmission [88] and choose diets for HIV-positive infants [89,90].

#### 4.2.2 Joint decision-making

In 27 % of the studies, decisions were made jointly by husband and wife, male elders/husband and in-laws/husband, husband and in-laws, husband, in-laws and health care providers or husband and female friends/neighbours/health care workers. In the human health context, joint decision-making is common among couples and often depends on women's decision-making power [23], their income [22] and the degree of economic dependence on men [69]. For example, husbands and wives jointly determine desired family size which is influenced by the contraception method a woman prefers and its perceived efficacy [21,22]. Another example involves disease-prevention decisions, such as allocating mosquito nets to available sleeping spaces. Couples allocate nets to individuals depending on their biological vulnerability to malaria, age and their economic position within the household [67]. During joint decision-making, women engage in bargaining, and their bargaining power tends to be stronger in urban than rural settings [91].

Joint treatment decisions are not limited to couples. For instance, when a child suffers from fever and convulsions, decisions are often influenced by advice from female friends, relatives and neighbours, alongside financial support from husbands and elders [68,92]. Sometimes, a husband and neighbour choose a pregnant woman's treatment, assessing illness severity to determine the type of care she needs, the time the care should be sought, the means of transport and how costs at the health facility will be covered [93]. Given limited resources and unequal decision-making power, wives may bargain by demonstrating good behaviour to husbands and by indirectly negotiating through seeking support from their mothers, brothers and uncles who can influence decisions in their favour [94].

Maternal health care decisions are also made jointly, often involving husbands, in-laws and traditional birth attendants (TBAs), and are constrained by household income for associated costs [95]. According to Gurara et al. [96], a TBA is a respected older woman with informally acquired home birth skills. Her main role is to assist a pregnant woman during the childbirth. Pregnant women trust TBAs because their care meets the community's cultural and traditional expectations. After birth, the woman's and newborn's care often reflects choices made by her husband and female friends and is constrained by the husband's financial resources [97]. Couples make joint decisions on nutrition, childcare, cleaning and cooking [98]. In some cases, they discuss and agree on nutrition and childcare both before and after childbirth [98].

Nutrition decisions are made jointly by husbands, wives, in-laws and health care providers. For instance, to prevent chronic energy deficiency, a husband and wife determine the diet-diversity preferences for household members [99]. Husbands, wives and mothers-in-law express their preferences on food acquisition, cooking and consumption at different phases of the food preparation [100]. When decisions concern diets for young children, health care providers, grandmothers and mothers choose the preferred food, while fathers provide the financial resources to obtain them [101].

#### 4.2.3 Institutional approach

The institutional approach demonstrates how the internal and external processes of the household interact during decision-making. The first component is social and it describes how religion, culture, norms and tradition influence decision-making around health. Some religious beliefs of the household determine whether a child is immunised or not [102]. For instance, Singh et al. [102] found out that women who practised Islamic religion were less likely to let their children go through the full immunisation schedule. The religious beliefs of the primary decision-maker also determine whether the choice of maternal health care provider is a modern medical facility or a traditional birth attendant [43]. This means that religion influences pregnant women's acceptance of delivering in health facilities [95,103].

Gender norms and intergenerational relations influence decisions over resource allocation [92]. For instance, it is often expected that a child's father will provide financial support for the treatment of a sick child based on his role as a father [83]. It is also expected that a wife will seek permission from her husband before accessing maternal health care [95]. Societal norms shape the community's perception of childhood illness and the period during which a child is considered a newborn [73]. These norms are important for determining the perceived implications of death and the value of the child based on birth order, gender and the priority given to allocate money for treatment [82]. Some societal norms also encourage communal decision-making [95] and membership to social networks with friends, neighbours and relatives from whom money is borrowed to cover health care costs [104]. Further, cultural beliefs shape carers' perceptions of a child's illness and whether it requires traditional or modern medicine treatment [83]. In some contexts, cultural beliefs also restrict pregnant women from delivering outside of the home, especially where exposure of women's bodies to male doctors is prohibited during childbirth [42]. These beliefs may strengthen trust in traditional birth attendants and practices before and after childbirth [43]. For instance, in pastoral communities of the Afar region of Ethiopia, childbirth is seen as a natural process deeply rooted in traditional and religious practices of the pastoralists' lifestyle [43]. Women are encouraged to give birth at home, because delivery at the health facility exposes their bodies to medical attendants which is prohibited, and could hinder the performance of prayers and rituals on the newborn immediately after birth [43].

The second component is the economic environment, comprising factors such as wealth, income, education and employment. For example, the purchase of mosquito nets to prevent malaria depends on the price of the net and the perceived benefits of using treated nets, including improvements in health outcomes for those using the nets and financial benefits for suppliers [105,106]. The number of nets bought depends on the average monthly income and forgone benefits resulting from malaria illness [107]. The allocation of financial resources for the treatment of infant illness depends on the cost of treatment [68] and the amount of money available to the father [73]. Other economic factors that determine the choice of treatment include the cost of consultation, the price of medicines, transport cost and waiting time at health centres [80].

The literacy level of primary decision-makers determines whether the household acquires a treated mosquito net or not in the southern part of Ethiopia [105]. The absence of a literate person in the household increases the likelihood of failing to seek health care, which can lead to under-five mortality [64]. More broadly, the education level of a caregiver partly determines whether a child is immunised or not, the choice of health care provider [108] and the choice of treatment [81]. It also influences the place of childbirth [89,109,110], family planning [91] and health investment decisions, such as acquiring health insurance [111,112].

Similarly, household wealth (assets) helps determine whether a child is delivered at home or in a health facility [43], whether a child is immunised or not [102] and whether they develop chronic malnutrition [113]. In some settings where men have formal employment and can exercise financial control, they play an important role in supporting women's access to contraceptive services [71] and in investing in health insurance [111]. However, in contexts where women have higher education, independent income or formal employment, couples are more likely to engage in joint decision-making around contraception and fertility [114].

The third component is the health system and its availability, which influence the type of health care sought. For instance, accessibility of a hospital is determined by distance from the household [107], as well as weather coupled with the quality of roads [20]. A well-equipped hospital and the attitude of hospital staff partly determine the acceptability of care [115] and the perceived quality of services [108] among household members. Other factors that influence the choice of hospital include the availability of medicines [81], severity of symptoms [68], timing of the decision to seek care [84], time spent at the hospital and financial resources available to meet basic needs such as food and housing versus enabling access to high quality health care [80].

The fourth component includes factors related to demographic and other characteristics of the individual and household that interact with the social, economic and health system environment during the decision-making. For example, in malaria prevention decisions, household size, age [67] and gender play an important role in the allocation of treated nets [105,106]. In maternal health care and neonatal decisions, age and ethnicity of the mother are important [64,102]. Decisions on contraception and fertility are influenced by a woman's marital status and attitude towards family planning [23].

### 4.3 The HPH framework in livestock health

#### 4.3.1 Sole decision-making

About 42 % of livestock-related decisions covering acquisition, management and health were made by a single person, predominantly (32 %) by men. Many of the studies (44 %) were exclusively carried out in rural areas where reliance on livestock is high. Sole decisions made by men often concern livestock ownership, health and investment choices. For example, men often select strategies to prevent vector-borne diseases in livestock [18] and purchase treatments for gastrointestinal nematodes in goats [6,116]. Other decisions made by men include investing in cattle insurance [117] and prioritising livestock health over human health and education [18]. Furthermore, men make strategic choices about the types of livestock to keep in the household, and these choices are determined by access to land, credit and market conditions [118].

Although fewer in number, some decisions are made by women, particularly regarding small livestock species such as poultry, pigs, sheep and goats. Women are identified as the key decision-makers in breed preferences and disease prevention for poultry, for example for Newcastle disease [[119], [120], [121]]. Women also make decisions related to calf care [18] and household milk consumption [116]. However, when women attempt to make market decisions independently, with the intention of generating cash from livestock sales, men often contest these decisions [122]. For example, in some pastoral communities, men limit migration patterns to areas far from the market to prevent their wives from selling milk to generate cash [123]. In such cases, women choose preferred livestock markets only when selling livestock to them as gifts by their fathers or husbands [124].

#### 4.3.2 Joint decision-making

Across livestock-related decisions, 32 % were joint, i.e. involving both men and women. However, the degree of joint decision-making varied by context, species and household structure. For example, during peak milk production, couples agreed on a milk sharing arrangement consisting of women controlling evening milk sales and men controlling morning sales [125]. When selecting a livestock breed, couples determined preferences for each livestock type based on traits such as expected product yield (e.g. milk), body size and colour, and its adaptability and ease of sale [127]. Although joint decision-making involves men and women, men often allocate and control the amount of decision-making power that women hold [127]. Moreover, the notion of ‘jointness’ is context-specific: in wealthier households, or where larger species such as cattle are involved, male dominance of decision-making increases, whereas women tend to have greater influence over decisions about smaller livestock such as poultry or goats [128].

#### 4.3.3 Institutional approach

Similar to decision-making around human health matters, here we identified four components within the institutional approach: social, economic, health system and demographic factors.

The social component comprises cultural values, norms and religious beliefs that govern livestock keeping and health decisions. Social benefits are derived from cultural practices, for example slaughtering livestock to feed visitors during holidays and wedding seasons, and mourners during funerals [122]. Further, the allocation of decision-making authority to women in the household can depend on a husband's symbolic capital [127]. Symbolic capital refers to the economic, social and cultural capital an individual possesses that legitimises claims to honour, respect, prestige, esteem and recognition [129]. In many settings, cultural norms assign most household decisions to men, with crop marketing being a common exception [130]. Women's ability to generate cash from livestock sales and participate in decision-making varies with culture [18,122]. In some communities (e.g. pastoralists), cattle keeping is considered a man's domain and the treatment of vector-borne diseases may therefore depend on his presence in the household, the supply of acaricides and his ability to self-treat sick cattle [18]. Culture also shapes the use of traditional medicines for livestock (e.g. Newcastle disease in poultry) which can, in turn, limit uptake of the respective vaccine [119].

Beyond culture, religion may also shape practices related to livestock handling and health. For instance, in Uganda, some studies report that Muslim, compared to Pentecostal poultry keepers were more likely to engage in risky practices such as irregular cleaning of water and feed troughs, potentially facilitating the spread of Avian Influenza [131].

The second (economic) component includes education, wealth, income and market factors that influence livestock health decisions. Education level influences enrolment rate into livestock insurance, transferring morbidity and mortality risk to a third party [117] and access to paid employment as a form of inheritance [132]. It also affects the choice of livestock breed [133], choice of a health provider and ability to adopt a breed, e.g. indigenous chicken [116,134]. Educated farmers are more likely than others to opt for open slaughter of birds to control avian influenza [131]. A farmer's ability to adopt artificial insemination depends on experience in dairy farming, record keeping and use of good management practices, such as water provision and feed availability [135].

Furthermore, household wealth can be reflected in ownership of large livestock [40], durable goods, farm equipment and large farm size [136,137]. In turn, wealth influences participation in vaccination programmes, e.g. against peste des petits ruminants [53] and the choice of health care provider [138]. In addition to wealth, income from livestock (e.g. chickens) sales depends on time and labour inputs [122] and on health investments [117]. Women generate cash from milk sales [123], influenced by the availability of market information, distance to the market and whether markets are local or urban [139,140].

The third component is the health system, characterised by factors such as illness type, availability and accessibility of livestock drugs and vaccines, drug quality [66] and type of health care provider available [138]. Other important factors include the distance to vaccination points [141] and source of livestock health information [29].

The fourth component comprises livestock demographic factors that, when considered alongside the social, economic and health system components, influence decision-making. For instance, the selection of livestock breed is determined by age, body condition, weight [139] and livestock type [40]. Animal sex is important in determining the number of cattle insured, production system employed [117] and the culling process applied [126]. The production system varies by scale, e.g. large, medium or small, and time [125].

### 4.4 The similarities, differences and synergistic connection between the human and livestock HPH frameworks

**Fig. 6:**
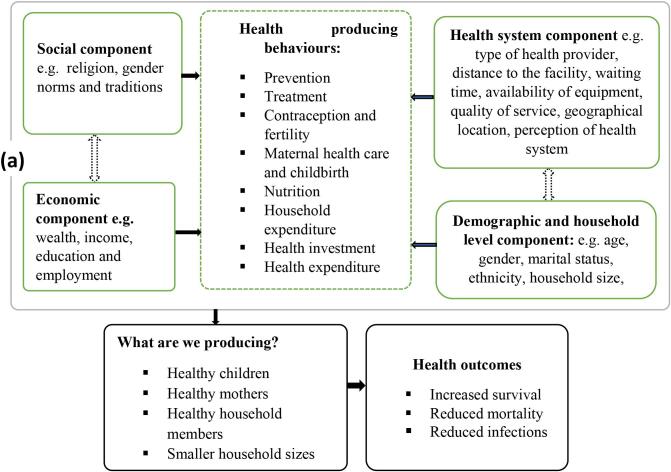

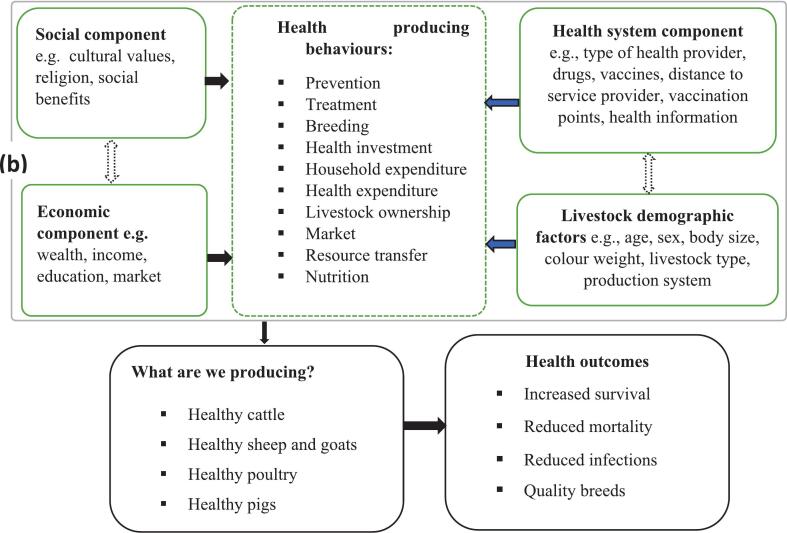
a: The HPH framework in human health (adapted from Berman et al. [27]). b: The HPH framework in livestock health (adapted from Berman et al. [27]).

To explore the relationship between the human and livestock HPH frameworks, we define the inputs required for the separate frameworks (Fig. 6a and b). We also specify the relationship between the two frameworks. Understanding both is necessary to design effective health interventions that improve overall household health outcomes.

In both the human and livestock health HPH frameworks, decision-making draws on common social, economic, health and demographic components that inform household health outcomes. Social factors such as cultural values, religion and social norms influence health decisions in both cases, with religious beliefs affecting choices of maternal health providers [95] and the use of traditional livestock medicines [119]. Economically, wealth, income, education and employment play a major role in determining health access and outcomes, as livestock ownership provides a financial buffer for human health expenditures, while financial constraints limit access to both human and animal health care services [137]. Health system factors, including distance to facilities, drug availability and health worker capacity, also matter, affecting both maternal health outcomes and livestock disease management [20,141]. Demographic and household-level characteristics, such as household size, gender roles and the age of decision-makers, shape how resources are distributed, with maternal care decisions often involving multiple family members and livestock health decisions being typically dominated by men [127].

More specifically, husbands, wives, in-laws, health care providers and neighbours are mainly responsible for decision-making in human health matters, while sick infants, children and pregnant mothers are the major beneficiaries of these decisions. Livestock health decisions are mainly made by husbands and wives on cattle, sheep, goats, poultry and pigs. For human health, important inputs include a healthy diet, quality medicines, contraceptives, vaccines, well-equipped hospitals, treated mosquito nets, income, wealth, cost of treatment, education and reduced waiting times at health facilities. Decision-makers draw on their skills, experiences, time, perception of disease severity, religious and cultural beliefs and the value they place on the beneficiaries to combine these inputs to attain desired health outcomes.

Similarly, livestock health decisions require inputs such as quality breeds, livestock markets, drugs, vaccines, vaccination points, artificial insemination, biosecurity measures and access to veterinary services. Livestock health decision-makers draw on their skills, time and understanding of the production system type, as well as, the animal's age, sex and other characteristics, to produce positive health outcomes [126]. Livestock ownership supports human nutrition and income, providing important protein for children and pregnant mothers [125,142], and serving as a form of wealth that can be converted into cash for medical expenses [137]. However, this dual role can create significant within-household resource trade-offs, as investing in livestock health may limit resources available for human health care and education [143].

Moreover, livestock ownership often confers within-household decision-making power, particularly to women, influencing their control over nutrition and income use [124]. The interconnected systems of human and livestock health create both synergies and within-household resource trade-offs, as livestock investments can support household well-being yet also constrain resources for human health, reinforcing existing gender dynamics and resource imbalances [40]. In both approaches, the household depends on both human and livestock health outcomes, generating utility and production-related within-household resource trade-offs. Livestock ownership can strengthen households' financial resilience in support of health, although livestock as an asset may also limit immediate access to human health care if animal health is not effectively managed.

## 5 Discussions, conclusions, limitations and future research recommendations

This review explores the complexities of decision-making around human and animal health matters in households across the SSA region. Using the HPH framework applied to human and livestock health separately, we analysed studies from eastern, central, western and southern Africa spanning over two decades. These studies examined various household decisions, from disease prevention and treatment to general health improvements (e.g. nutrition and breeding choices in livestock) and how households allocate resources to address specific health concerns.

In the studies we reviewed and analysed, sole decisions comprised the majority category (40 % in human health and 42 % in livestock health). Joint decision-making was less common, occurring in 27 % of human health and 32 % of livestock health-related cases. Usually, men, particularly male household heads, were the sole decision-makers, whereas joint decisions often involved spouses or extended family including in-laws. Women frequently had to request permission to access male-controlled finances to pay for treatment or care, even when they were directly managing it. These patterns highlight the influence of gender roles on human and animal health outcomes. In SSA, livestock contribute to improved health by providing nutrition, income and cash for education and medical care. Although women are central to subsistence economies and livestock management, their role in livestock decision-making remains constrained by sociocultural barriers. As a result, One Health interventions must explicitly consider these gender-based power dynamics to be effective.

Building on Berman et al.'s [27] HPH framework, our study incorporates the social, economic and health system-environment factors related livestock health. This expanded approach enabled us to examine how decisions regarding human and animal health are influenced by overlapping factors such as gender norms, educational attainment, household income and access to key resources. For example, joint decision-making was more prevalent in families where women had formal education or paid employment, whereas male-dominated sole decision-making was more common in traditionally structured households where men controlled financial assets. Where these empowering conditions were absent, women were often expected to conform to ‘socially acceptable behaviour’ to gain approval from husbands or in-laws. These findings build on previous research addressing gendered disparities in health-related decision-making [e.g. 43,94,95] and extend them to the livestock sector, which has received less attention. Additionally, health system-related factors such as access to livestock vaccines, distance to facilities and perceptions of provider competence also shape household decisions, often affecting whether health seeking care is delayed or avoided altogether.

Our analysis reinforces the idea that household decision-making in SSA households often resembles Becker's [144] “black box” in which male dominance conceals the internal dynamics of negotiation. However, joint decision-making introduces flexibility within this system. High rates of sole decision-making reflect a system that often ignores individual household members' preferences. In the sole-decision models, one individual, typically the male head, controls income and consolidates it into a single household budget. Several studies in our review show that decisions regarding child health and vaccinations often require male approval, restricting access and revealing an understated but significant pattern of intra-household negotiation [42]. Even when processes appear decentralised, women's reliance on male-controlled finances indicates a hidden struggle to access health care [145]. In many cases, women must formally request permission to use household resources, effectively casting men as the gatekeepers of financial power. This structure allows male authority to mask internal bargaining, with financial decisions made unilaterally and the internal negotiation process hidden in plain sight [146,147].

Addressing these inequalities requires an acknowledgement of the social and cultural frameworks that shape them. Although shared decision-making tends to yield better health results and promote fairness, it cannot simply be mandated through direct shifts in control, as this may conflict with prevailing social norms. Efforts to shift authority from men to women without addressing deeper structural issues can provoke social resistance. A more effective strategy involves fostering cooperative decision-making that values the contributions of both partners. Practical steps might include joint budgeting and transparent communication about finances as well as encouraging male household members to support shared authority. One useful approach is to implement community programmes that actively involve both men and women in discussions about resource allocation, especially concerning maternal health, nutrition or livestock care. Furthermore, allowing women more decision-making power in areas where they already hold practical responsibilities, such as child health or food choices, while promoting dialogue within the household, can improve both the quality of decisions and overall health outcomes.

Taken together, these findings translate into clear, actionable steps: design gender-responsive One Health programmes that (i) co-create interventions with couples and influential kin, (ii) pair maternal/child services with accessible veterinary outreach and (iii) use simple joint-budgeting and decision tools to make resource trade-offs explicit. To summarise, household decisions related to both human and animal health in the SSA region are influenced by the interplay of gender roles, demographic, economic factors and cultural traditions. While men commonly make decisions alone, greater involvement of women, especially when supported by formal education, income generation or employment, can lead to more shared decision-making. Achieving fairness in household decision structures calls for sustained efforts in gender-inclusive policymaking, as well as strengthening education and health care systems. The most effective approaches are those that encourage joint responsibility, empower women in areas where they already hold practical authority and engage men as active partners in household wellbeing. Moreover, broader strategies, like expanding universal health coverage and improving access to both medical and veterinary services are critical for easing the pressures of internal family negotiations and building healthier, more resilient households. Furthermore, co-designing community-level health and outreach programmes that encourage the involvement of both partners, including introducing them to household budgeting tools, may promote transparency in decision-making.

This review has several important limitations. Firstly, by focusing only on English-language sources, we may have inadvertently emphasised East African contexts, since French is more commonly used in West and central Africa. Additionally, the higher number of livestock in East Africa (98,524 Tropical Livestock Units (TLUs)) relative to West (53,016 TLUs), southern (16,731 TLUs) and central Africa (9843 TLUs) likely contributed to this geographic imbalance. According to Nthambi et al. [148], a TLU is a standard measure of livestock holdings. Secondly, most studies used qualitative, quantitative and mixed-method approaches making it difficult to statistically pool results together without potentially giving misleading results when quantifying inequalities or formally testing household decision-making modes across contexts. Using quantitative methods alone might have enabled a meta-analysis to measure how different decision-making practices contribute to disparities. Thirdly, while we aimed to generalise our findings across the SSA region, cultural, economic and health-system variables vary markedly by region, potentially altering their interactions. Lastly, reliance on secondary literature limited our ability to rigorously evaluate unitary versus collective decision-making models within the HPH framework. Consequently, this review provides a descriptive rather than a statistically driven analysis of decision-making dynamics.

Because our review was limited to studies published on or before May 2020, future research should incorporate the upcoming post-pandemic literature on household decision-making and its implications for One Health applications. The COVID-19 disrupted health care access and financing and may have affected household decision-making, especially through shifting intra-household bargaining power [149], with important implications for the design and implementation of One Health policies. To build a stronger evidence base, upcoming reviews should combine qualitative mapping of intra-household bargaining with quantitative measurements of decision locus and health/livelihood outcomes, using mixed-method designs and longitudinal or panel data to track changes over time. Leveraging large, gender-sensitive datasets (e.g. DHS for human health; WEAI, pro-WEAI and WELI for agriculture/livestock) will enable comparative analyses across settings and species and allow rigorous tests of whether shared decision-making improves resilience.

## CRediT authorship contribution statement

**Mary Nthambi:** Writing – review & editing, Writing – original draft, Visualization, Validation, Methodology, Formal analysis, Data curation, Conceptualization. **Tiziana Lembo:** Writing – review & editing, Supervision, Project administration, Funding acquisition, Conceptualization. **Alicia Davis:** Writing – review & editing, Project administration, Funding acquisition, Conceptualization. **Blandina T. Mmbaga:** Writing – review & editing, Project administration, Funding acquisition. **Nicholas Hanley:** Writing – review & editing, Supervision, Methodology, Funding acquisition, Conceptualization.

## Funding

We acknowledge the financial support by the Antimicrobial Resistance Cross-Council Initiative through a grant from the Medical Research Council, a Council of UK Research and Innovation, and the National Institute for Health Research (MRC/AMR/ MR/S004815/1).

## Declaration of competing interest

The authors declare that no personal relationships and or commercial interests exist that could affect the findings of this manuscript. Moreover, we confirm that no financial support or information extraction permissions may directly or indirectly raise concerns around the findings in this manuscript. All authors have reviewed and approved the content of this manuscript and agree with the criteria used to determine the authorship list.

## Appendix A Supplementary data

Supplementary material 1: Mathematical formulation of the HPH framework. Supplementary material 1

Supplementary Material 2: Summary of studies reviewed. Supplementary material 2
