# Supplementary material for: Understanding decision-making around human and livestock health in sub-Saharan Africa: A systematic literature review
Source: Dialogues Health. 2025 Dec;7:100259. doi: 10.1016/j.dialog.2025.100259 (PMC12704076; doi:10.1016/j.dialog.2025.100259)
Supplement: Supplementary file 1 — Supplementary material 1: Mathematical formulation of the HPH framework. [file mmc1.docx]

**Supplementary material 1: Mathematical formulation of HPH framework**

To understand further how health in the household is produced, let us assume that consumers demand ‘good health’ outcomes and not medical services *per se* and that they produce these health outcomes using inputs such as medicines, healthy diets and clean water amongst others (Grossman, 1972).

An example is a pregnant mother in the household who aims at delivering a healthy infant. If the infant’s body weight at birth is used as an indicator of good health, we can describe two utility functions. The first relates to the household’s demand for goods that affect the infant’s health, while the second arises from the benefit the infant gains from consuming the good (Mwabu, 2009). The utility function of the infant is entrenched in the utility maximising behaviour of the mother (Mwabu, 2009). Based on Mwabu (2009), and Rosenzweig and Schultz (1983)’s production of health model, we express the two utility maximisation functions as follows:

$$U=U \left( K,Y,A \right) (1)$$

where U represents a household’s demand which is a function of three main properties: (1) a neutral good K which does not directly contribute to the health outcome of the infant, for example clothes for the mother or other members of the household; (2) a health-related good, e.g. medical care, recreation, diet, exercise etc, or behaviour, e.g. consuming alcohol and smoking (which negatively affects the birth weight) (Y); and (3) the health status of the foetus in the womb (A) which can be expressed using production function (2).

$$A=M\left( Y, X,\mu\right) (2)$$

where X represents expenditure on inputs such as medicines, food and medical services etc, Y is the health-related good described above and $\mu$ represents known environmental or genetic factors but is not determined by the behaviour of the pregnant mother. The utility expressed in equation 1 is maximised subject to the production function in (2) and a budget constraint S (eq. 3) (Rosenzweig and Schultz, 1983).

$S= {KP}_{k}+ {YP}_{y}+{XP}_{x} (3)$

where S represents household income and $P_{k}$, $P_{y}$ and $P_{x}$ are the prices for the non-health good K, health-related consumer good Y and infant investment good X, respectively. The infant investment good is assumed to have been purchased to improve the health of an infant and it enters the mother’s utility function through A. Each of these goods can be expressed separately to yield health input demand functions (see Mwabu, 2008).

Let us assume that the household has a husband (h) and a pregnant wife (w), but the husband is the dominant decision-maker on the purchase of the health-related good Y. To purchase Y, the husband pools household income and resources and determines the specific quantities of the said good. Each individual husband (h) and wife (w) receives a certain level of satisfaction from the purchase of good Y. When the good is consumed by the wife (w) and foetus (i) the unitary utility function maximised by the household is defined as:

$U\left( Y^{w},Y^{i} \right) (4)$

where $Y^{w}$ and $Y^{i}$ represent the consumption of good Y by the pregnant wife (w) and infant (i), respectively. The individual utilities of the wife and foetus are maximised in a single function subject to a budget constraint:

$p\left( Y^{h}+ Y^{w} \right)=k=k^{h}+ k^{w} (5)$

where $k^{h}$ and $k^{w}$ represent husband’s and wife’s incomes which are pooled together to form total income (k) (Lundberg and Pollak, 2008). The resulting household demand function for good Y is expressed as $Y^{n}=f^{n}(p, k)$ which is determined by prices (p) and family incomes (k) (Lundberg and Pollak, 2008).

However, if husband (h) and wife (w) make decisions together on the purchase of good Y, we express the household utility function as follows (Browning et al., 2006):

$$u\left( k, Y;z, c,\theta\right)={max}_{y^{H},y^{w}}\left\{ u\left( z,\theta\right)V^{h}\left( y^{h},Y;c \right)+\left( 1-u\left( z,\theta\right) \right)V^{w}\left( y^{w},Y;c \right) \right\} \left( 6 \right)$$

where u is the utility being maximised, z are the factors determining the distribution of decision-making power, c are the individual specific factors such as age, gender and education of decision-makers, and household characteristics such as household size and location (Browning et al., 2006), and$u\left( z,\theta\right)$ represents the efficiency weight which ranges between zero (0) and 1 and determines the influence of husband on the demand of good Y. If $u=1$, the husband (h) is an effective dictator, while when $u=0$ the wife(w) becomes the dictator (Browning et al., 2006). The utility maximisation function (6) is subject to a budget constraint.

$k=k^{h}+ k^{w} (7)$

where k is income and, $k^{h}$ and $k^{w}$ represent the husband’s and wife’s share of income, respectively.

Another example in livestock health can be described with a slight modification of the model in Amini-Rarani et al. (2019), which we adapt in our case to express the health function for livestock. In this example, we use mortality rate of calves as an indicator of health status of calves owned by the household. A lower mortality rate, which is estimated as a ratio of number of calves that died versus those that are being reared, represents a good health status, while a higher mortality rate is an indicator of poor health (Depoorter et al., 2015). However, the institutional approach accounts for the external processes of the household by describing how the demographic factors interact with the social, economic and health systems to influence health production and consumption decisions (Berman et al., 1994; Tipper et al., 2010). From an institutional approach, calf mortality (CM) is determined by four main factors that include the primary decision maker’s socioeconomic factors (DS), the cow’s (C) and the calf demographic (CD) factors and the health system (HS) factors. The function is expressed as follows:

$$CM=f\left( DS, C, CD, HS \right) (8)$$

**References**

Grossman, M., 1972. On the concept of health capital and the demand for health. Journal of Polical Economy, 80(2), 223-255.

Mwabu, G., 2009. The Production of Child Health in Kenya: A Structural Model of Birth Weight. J. African Economies,

Rosenzweig, M.R., Schultz, T.P., 1983. Estimating a Household Production Function: Heterogeneity, the Demand for Health Inputs, and Their Effects on Birth Weight. J. Pol. Econ., 91(5), 723-746.

Lundberg, S., Pollak, R.A., 2008. Family Decision Making. Palgrave Macmillan (ed.), The New Palgrave Dictionary of Economics.

Browning, M., Chiappori, P., Lechene, V., 2006. Collective and unitary models: A clarification. Rev Econ Household, 4, 5–14.

Depoorter, P., Huffel, X.V., Diricks, H., Imberechts, H., Dewulf, J., Berkvens, D., Uyttendaele, M., 2015. Measuring general animal health status: Development of an animal health barometer. Preventive Veterinary Medicine, 118(4), 341-350.
